# Gastrointestinal tolerance to a standardized milk-based hydration strategy is similar across exercise modalities

**DOI:** 10.3389/fnut.2026.1800364

**Published:** 2026-04-23

**Authors:** Julián Camilo Garzón-Mosquera, Priscilla Portuguez-Molina, Luis Fernando Aragón Vargas

**Affiliations:** 1 Human Movement Science Research Center (CIMOHU-UCR), University of Costa Rica (UCR), San José, Costa Rica; 2 School of Physical Education and Sports, University of Costa Rica (UCR), San José, Costa Rica

**Keywords:** endurance exercise, exercise modality, gastrointestinal symptoms, hydration strategy, lactose-free A2 milk

## Abstract

**Background:**

Gastrointestinal (GI) symptoms are frequently reported during endurance exercise, and running is often assumed to provoke greater GI discomfort than cycling due to higher mechanical impact. However, most supporting evidence derives from field studies with limited control of exercise intensity, hydration volume, and beverage composition, making it difficult to isolate the independent effect of exercise modality. This study aimed to compare GI symptom burden and beverage palatability between treadmill running and stationary cycling under controlled conditions.

**Methods:**

In a randomized crossover design, physically active adults completed treadmill running and stationary cycling under standardized conditions, including matched relative exercise intensity, duration, hydration volume, and beverage composition. A low-fat, lactose-free A2 cow’s milk was used as the sole hydration beverage. GI symptoms were assessed repeatedly throughout exercise, and beverage palatability was evaluated using hedonic ratings.

**Results:**

Overall GI symptom burden was comparable between modalities, with no significant differences in aggregate upper GI, lower GI, or systemic scores (all *p* ≥ 0.34), and equivalence confirmed for upper GI and systemic regions. Although cycling was associated with higher odds of heartburn/acidity (OR = 7.40; *p* = 0.001), flatulence (OR = 2.45; *p* = 0.012), defecation urgency (OR = 231.40; *p* = 0.007), and headache presence (OR = 2.38; *p* = 0.044), these differences did not translate into higher composite symptom burden. Palatability remained acceptable and did not differ between modalities.

**Conclusion:**

When exercise intensity, duration, hydration volume, and beverage composition are controlled, exercise modality does not meaningfully influence GI tolerance. Low-fat, lactose-free A2 milk was well tolerated across both modalities, supporting its use as an in-exercise hydration beverage regardless of exercise mode.

## 1 Introduction

Gastrointestinal (GI) symptoms are among the most commonly reported complaints during endurance exercise, with prevalence estimates from field studies ranging from about one quarter to nearly all participants (≈25–94%), depending on how symptoms are defined, the event duration, and the exercise modality (1–5). These symptoms—including nausea, abdominal cramping, bloating, and urgency—can impair performance, limit training adherence, and occasionally force withdrawal from competition (3, 4). Critically, the incidence and distribution of GI distress appear to vary across exercise modalities. Running, in particular, has repeatedly been associated with a higher frequency of GI complaints than cycling: long-distance runners report a higher burden of lower-tract symptoms (≈71%) than upper-tract symptoms (≈36%), whereas cyclists tend to experience both upper and lower GI problems at similar frequencies (≈64–67%) (3). Triathletes also report a high prevalence of GI complaints (up to 94% with at least one symptom) and particularly frequent lower-tract symptoms during the running segments (2, 3). Such modality-specific differences have important practical implications: athletes who transition between sports, coaches designing cross-training programs, and practitioners developing hydration and fueling guidelines all require evidence-based information on how exercise mode influences tolerance to fluid and nutrient intake during exertion.

The biomechanical disparities between running and cycling have been proposed as plausible mechanisms underlying differential GI responses during endurance exercise. Running, as a weight-bearing activity, involves repetitive impacts and greater vertical displacement, which may promote increased abdominal organ movement and mechanical stress on the gastrointestinal tract, in contrast to the smoother motion observed in cycling (6). By contrast, cycling is predominantly non–weight-bearing, with minimal impact forces and reduced vertical displacement, and has often been associated with a lower frequency and severity of several GI symptoms compared with running in triathletes and endurance athletes (1–3). These mechanical differences have been suggested to influence gastric emptying, splanchnic blood flow redistribution, and epithelial barrier integrity—physiological processes implicated in exercise-induced GI dysfunction (5). In addition, the postural and ventilatory constraints inherent to running may limit fluid intake to smaller, intermittent volumes, whereas cycling generally allows the consumption of larger boluses at more regular intervals. However, despite these well-recognized biomechanical and practical distinctions, direct experimental comparisons of GI tolerance between running and cycling under tightly matched physiological and nutritional conditions remain scarce. Much of the available evidence derives from separate studies employing heterogeneous exercise protocols, beverage compositions, and relative intensities, thereby limiting the ability to isolate the independent effect of exercise modality on GI responses.

Direct experimental evidence does not consistently support the assumption that running causes greater intestinal injury than cycling. Edwards et al. (7) directly compared objective intestinal damage—assessed via plasma intestinal fatty acid binding protein (I-FABP), a sensitive biomarker of enterocyte injury—during 45 min of running and cycling at matched absolute oxygen uptake (70% V̇O_2_max). Contrary to expectations, the magnitude of I-FABP change was significantly greater following cycling than running (+ 84.7% vs. + 19.3%; *d* = 0.68, *p* = 0.024), accompanied by higher RPE and heart rate during cycling and no difference in rectal temperature between modes. The authors concluded that physiological exercise intensity, rather than modality-specific mechanical stress, is the primary determinant of exercise-induced intestinal damage. Importantly, however, Edwards et al. (7) assessed mucosal injury via a circulating biomarker rather than perceived GI symptoms, and neither that study nor Costa et al. (5) evaluated GI tolerance to a milk-based hydration strategy under standardized nutritional intake conditions—the gap the present study addresses. Because perceived GI symptoms, rather than biochemical markers of intestinal injury, determine whether a hydration strategy is practically tolerable during exercise (4, 8), symptom-based assessment represents the primary athlete-relevant outcome for this research question.

A growing body of evidence has renewed interest in milk-based beverages as viable hydration strategies in sport, given milk’s integrated nutritional matrix of water, electrolytes (Na^+^, K^+^), carbohydrate, and protein, which has been proposed to support fluid balance and recovery (9). In controlled post-exercise rehydration trials, milk-based beverages have demonstrated greater fluid retention than water and, in some cases, higher retention than carbohydrate–electrolyte sports drinks. For example, Desbrow et al. (10) reported significantly greater fluid retention with cow’s milk compared with a commercial sports drink following exercise-induced dehydration. Similarly, Aragón-Vargas et al. (11) showed that skimmed lactose-free milk produced greater fluid retention and a more favorable net fluid balance than water in a post-exercise rehydration protocol, with the sports drink generally yielding intermediate responses. The Beverage Hydration Index (BHI), developed by Maughan et al. (12) under resting conditions in euhydrated participants, further demonstrated that both skimmed and full-fat milk elicited lower urine output and higher BHI values than water over a 4-h observation period, ranking among the beverages with the greatest fluid retention capacity in that protocol. During exercise, Aragón-Vargas et al. (13) investigated voluntary intake of skimmed lactose-free milk during 90 min of moderate-intensity cycling in the heat and found that, despite lower voluntary consumption compared with water, milk maintained a comparable net fluid balance and hydration status, with only mild GI symptoms reported. Emerging evidence further indicates that gastrointestinal tolerance to milk is influenced not only by lactose content but also by protein composition, particularly β-casein variants, with A2-based and lactose-free formulations generally associated with reduced GI symptoms and good overall tolerance in sensitive individuals (14, 15).

Collectively, these findings support milk-based beverages as effective hydration strategies in both post-exercise and controlled in-exercise settings, likely due to their integrated matrix of water, electrolytes (Na^+^, K^+^), carbohydrate, and protein (9). Post-exercise studies and Beverage Hydration Index (BHI) data show that milk promotes greater fluid retention than water and often comparable or superior responses to sports drinks (10–12). During exercise, skimmed lactose-free milk has been shown to maintain euhydration as effectively as water during 90 min of moderate-intensity cycling, despite lower intake volumes (13); however, most evidence derives from low-impact modalities, leaving uncertainty regarding gastrointestinal tolerance and acceptability during higher-impact activities such as running.

The protein and residual fat content characteristic of dairy beverages could, in theory, interact with the mechanical stressors of running by influencing gastric emptying or exacerbating upper GI symptoms under impact conditions. Conversely, emerging evidence suggests that when exercise intensity and duration are carefully matched, differences in overall GI symptom burden between running and cycling may be smaller than early field observations imply (5). To date, this possibility has not been examined using milk-based hydration strategies under standardized exercise and intake conditions.

Despite the widespread assumption that exercise modality—particularly the higher mechanical impact associated with running—exerts a meaningful influence on GI tolerance, direct experimental evidence under controlled conditions remains limited. Most comparative studies have been conducted in field settings, where differences in exercise intensity, hydration volume, and beverage composition may confound modality-specific effects. Therefore, this study aimed to compare GI symptom burden and beverage palatability between treadmill running and stationary cycling at matched intensity using low-fat, lactose-free A2 milk as the sole hydration beverage, to determine whether exercise modality independently influences GI tolerance under standardized nutritional and physiological conditions. Based on the higher prevalence of GI complaints in runners and exploratory evidence of upper GI disturbances during cycling (13), we hypothesized that (1) treadmill running would elicit greater GI symptom severity than cycling, particularly for upper GI symptoms, and (2) palatability would remain acceptable in both modalities.

## 2 Materials and methods

### 2.1 Study design

**FIGURE 1:**
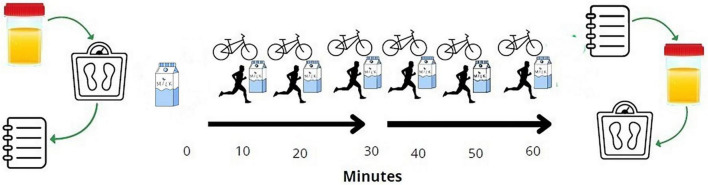
Experimental protocol. Participants arrived fasted (4–6 h), provided a urine sample, and had body weight measured. Baseline GI symptoms were assessed upon arrival. 10 min before exercise, participants ingested a pre-exercise bolus of 4 mL⋅kg^–1^ of lactose-free A2 milk (12°C), followed by pre-exercise GI symptom and palatability assessments. Participants then completed 60 min of continuous exercise on a cycle ergometer or treadmill (randomized order) at 70–80% of age-predicted HRmax. During exercise, 2 mL⋅kg^–1^ of A2 milk was ingested every 10 min (at 10, 20, 30, 40, 50, and 60 min), yielding a total intake of 16 mL⋅kg^–1^. Exercise was paused for 3 min at 30 min for GI symptom and palatability evaluation. A final GI symptom and palatability evaluation was completed immediately post-exercise, followed by urine collection and body weight measurement.

A randomized, controlled crossover design was employed to compare GI tolerance and palatability between two exercise modalities [treadmill running (T) and stationary cycling (C)] performed at matched relative intensity (Figure 1). Each participant completed both conditions in randomized order, separated by at least 48 h to ensure full recovery. The crossover design maximizes statistical power by using each participant as their own control, thereby minimizing inter-individual variability in GI sensitivity, aerobic fitness, and beverage preferences. All participants provided written informed consent prior to participation, and the study protocol was approved by the Ethics and Science Committee of the University of Costa Rica (approval number: CEC-654-2023), in accordance with the Declaration of Helsinki.

### 2.2 Participants

Twenty-eight physically active men and women aged 18–40 years were recruited from the university community and local athletic clubs. Inclusion criteria required: (1) regular participation in endurance exercise (≥ 3 sessions per week, ≥ 30 min per session) for at least 6 months prior to enrollment; (2) familiarity with both C and T exercise; and (3) no history of lactose intolerance or adverse reactions to bovine milk consumption. Prior to the first session, the Physical Activity Readiness Questionnaire (PAR-Q) was administered to assess physical readiness and screen for contraindications to exercise testing (16).

Exclusion criteria included: (1) diagnosed metabolic, GI, or cardiovascular diseases; (2) current use of medications affecting hydration status or digestive function (e.g., diuretics, laxatives, antihistamines, proton pump inhibitors); (3) pregnancy or lactation; (4) inability to attend both testing sessions within a 2-week period; and (5) body mass index < 18 or > 30 kg/m^2^. All 28 enrolled participants completed both exercise conditions with no dropouts or adverse events.

### 2.3 Beverage

The hydration beverage was a commercially available low-fat, lactose-free A2 milk containing exclusively β-casein A2 protein variant (Dos Pinos^®^). This formulation was selected to minimize potential GI distress associated with lactose intolerance or A1 β-casein sensitivity while preserving the electrolyte and protein content characteristic of dairy beverages. Nutritional composition per 250 mL serving: 8 g protein, 5 g fat, 12 g carbohydrate, 125 mg sodium, and 375 mg potassium (osmolality ∼280–300 mOsm/kg). The milk was stored under refrigeration (4°C) and served at 12°C to enhance palatability during exercise. No other food or beverages were permitted during the experimental sessions.

### 2.4 Pre-testing standardization

To minimize confounding effects of prior dietary intake and hydration status, participants received standardized verbal and written instructions for the 24 h preceding each trial. Participants were instructed and verbally confirmed adherence to similar food and fluid intake prior to each experimental session. Pre-trial guidelines required participants to: (1) consume at least five additional 250 mL glasses of water beyond habitual intake; (2) avoid alcohol, highly sweetened beverages, and known GI irritants (e.g., spicy or high-fiber foods); (3) maintain habitual caffeine intake without modification; (4) avoid diuretics, laxatives, and antihistamines; and (5) refrain from strenuous exercise (> 75% estimated HRmax or > 60 min) during the 24 h before testing.

On test days, participants arrived at the laboratory after a minimum of 4 h and no more than 6 h since their last meal to ensure partial gastric emptying while avoiding extreme hunger. Compliance with pre-trial instructions was verbally confirmed upon arrival.

### 2.5 experimental protocol

Upon arrival, participants changed into lightweight athletic clothing and provided a mid-stream urine sample for determination of urine specific gravity (USG) using a handheld refractometer (ATAGO^®^ URC-Ne, Tokyo, Japan; measurement range 1.000–1.050, precision ± 0.001). Euhydration was confirmed using a cutoff of USG ≤ 1.020 (17); no participants exceeded this threshold. Nude, dry body weight was then measured to the nearest 0.1 kg using a calibrated digital scale (e-Accura^®^ DSB291, Shanghai, China) after towel-drying and in a private area. Standing height was measured to the nearest 0.5 cm using a wall-mounted stadiometer (SECA^®^ 286, Hamburg, Germany).

At baseline (upon arrival), participants completed a previously published 16-item Likert-scale GI symptom scale (13). The scale employs a Likert scale ranging from 0 (no problem) to 9 (the worst it has ever been) and assesses 16 symptoms across three anatomical sections:

1. Upper GI tract (7 symptoms, max score 63): belching, heartburn/acidity, nausea, reflux, thickened or “cut” saliva, vomiting, “CRAMPS”
2. Lower GI tract (5 symptoms, max score 45): abdominal distention, abdominal pain (right or left side), defecation urgency, flatulence, intestinal cramps
3. Systemic (4 symptoms, max score 36): dizziness, headache, muscle cramps, urinary urgency.

Ten minutes before starting exercise (pre-exercise time point), participants ingested an initial bolus of 4 mL/kg body weight of A2 milk and completed the GI symptom scale for a second time. At this time point, they also completed a 9-point hedonic scale to rate the palatability of the milk (1 = extremely unpleasant, 9 = extremely pleasant) (18).

Participants performed 60 min of continuous exercise on either a stationary bike (Schwinn AC Performance Plus, Vancouver, WA, United States) or a treadmill (Cosmed^®^ T170, Cologne, Germany). The order of modalities was determined by simple randomization (coin flip) prior to the first session. Exercise intensity was standardized at 70–80% of age-predicted maximum heart rate, calculated using the equation HRmax = 207 − (0.7 × age) (19). This intensity range was selected because it corresponds to the moderate-to-vigorous aerobic category as defined by physical activity guidelines (20), has been applied in analogous exercise research to classify endurance intensity via %HRmax (21, 22), and is consistent with prior in-exercise hydration research employing this prescription approach (13). Specifically, this range was designed to be sufficiently demanding to induce meaningful physiological stress and potentially exacerbate GI symptoms, while remaining below the anaerobic threshold in most participants to minimize the confounding influence of metabolic acidosis and excessive sympathetic activation on GI motility. For the sample’s mean age (25.5 ± 5.0 years, estimated HRmax ≈ 189 bpm), this range corresponds to approximately 132–151 bpm, encompassing the observed mean HR values for both modalities. Although HRmax-based prescription does not fully account for inter-individual variation in aerobic fitness, the crossover design minimized this concern by ensuring each participant served as their own control across both modalities. As an adjunct intensity control, participants were verbally instructed to maintain a perceived exertion between 12 and 15 on Borg (23) 6–20 scale (corresponding to “somewhat hard” to “hard”), and compliance was verbally verified by the supervising researcher throughout each session. The selected 70–80% HRmax range was used as a practical, field-applicable method of standardizing exercise intensity, given the established approximately linear relationship between HR and V̇O_2_ across a wide range of submaximal intensities, while acknowledging that the HR–V̇O_2_ relationship is individual and can be influenced by external and physiological factors (21).

Heart rate was monitored continuously using a chest-strap heart rate monitor (Polar^®^ Pacer, Kempele, Finland) and recorded at 10-min intervals throughout exercise. During exercise, participants ingested 2 mL/kg body weight of A2 milk every 10 min (at 10, 20, 30, 40, 50, and 60 min), for a total in-exercise intake of 12 mL/kg. Combined with the pre-exercise bolus, total fluid intake was 16 mL/kg body weight over the 70-min period (e.g., 1,120 mL for a 70 kg individual, providing approximately 35.8 g protein, 22.4 g fat, and 53.8 g carbohydrate). Fluid boluses were consumed within approximately 30–60 s while participants continued exercising at the prescribed intensity, with no reduction in treadmill speed or cycling cadence during drinking. At 30 min, exercise was paused for 3 min to allow participants to complete the GI symptom scale and hedonic scale without motion artifact or attentional interference. This was the only pause in exercise and was applied identically across both modalities. Immediately after completing 60 min of exercise, participants dismounted the equipment and completed the GI symptom scale and hedonic scale for the final time. They then fully emptied their bladders into a pre-weighed container, with urine volume determined gravimetrically to the nearest 1 g using a digital balance (OHAUS^®^ CS2000, Parsippany, NJ, United States). After towel-drying, final nude body mass was measured using the same procedures as at baseline.

Ambient temperature and relative humidity were monitored continuously using a digital heat stress monitor (Questemp36^®^ 3 M, Oconomowoc, WI, United States) positioned at equipment height. Laboratory conditions were stable at 23–26°C dry bulb and 60–70% relative humidity throughout all testing sessions.

### 2.6 Statistical analysis

Descriptive statistics (mean ± SD, median, interquartile range) were calculated for all variables. Normality was assessed using Shapiro-Wilk tests (urine output, initial USG, and mean heart rate (*p* > 0.05)) and visual inspection of Q-Q plots. Homogeneity of variances was evaluated using Levene’s test (all *p* > 0.05, ranging from *p* = 0.126 to *p* = 0.944).

Physiological variables were analyzed using linear mixed-effects models (LMMs) with exercise modality (T vs. C) as a fixed effect and participant as a random intercept to account for within-subject correlation. Pairwise comparisons between modalities were performed using Wilcoxon signed-rank tests for non-normally distributed variables. Multiple comparison corrections were applied using the False Discovery Rate (FDR) method.

GIS were for section-level aggregated scores (upper GI, lower GI, systemic), cumulative link mixed models (CLMMs). Individual symptoms were analyzed using tailored strategies based on their prevalence and distribution: binary logistic regression for presence/absence of infrequent symptoms, ordinal CLMMs for symptoms with moderate prevalence, and hurdle models for frequent but zero-inflated symptoms. Hedonic ratings were analyzed using generalized linear mixed-effects beta regression models. All four assessment time points (baseline, pre-exercise, 30 min, and end of exercise) were included in the inferential models, with baseline serving as the reference category for temporal comparisons.

*Post hoc* power analyses (*n* = 28, α = 0.05) indicated ∼86% power to detect a standardized effect size of 0.6 SD (Cohen’s d), corresponding to a medium-to-large difference in GI symptom scores between modalities. Equivalence between T and C was assessed using Two One-Sided Tests (TOST) with equivalence bounds of ± 0.6 SD (Cohen’s *d* = 0.6), following the smallest effect size of interest (SESOI) approach recommended by Lakens (24). In the absence of a formally validated minimal clinically important difference for exercise-induced GI symptom scales, the equivalence bound was set to the smallest effect size the study had sufficient power to detect given the available sample size (*n* = 28, α = 0.05, ∼86% power at *d* = 0.6). Equivalence was concluded when the 90% confidence interval for the mean difference lay entirely within these bounds.

All analyses were conducted using R software (version 4.3.0) within RStudio. Statistical significance was set at *p* < 0.05 for all tests.

## 3 Results

Twenty-eight physically active participants (22 males, 6 females) completed the study, with a mean age of 25.5 ± 5.0 years, body weight of 67.3 ± 11.0 kg, and height of 169.6 ± 7.6 cm.

### 3.1 Gastrointestinal symptoms

**FIGURE 2:**
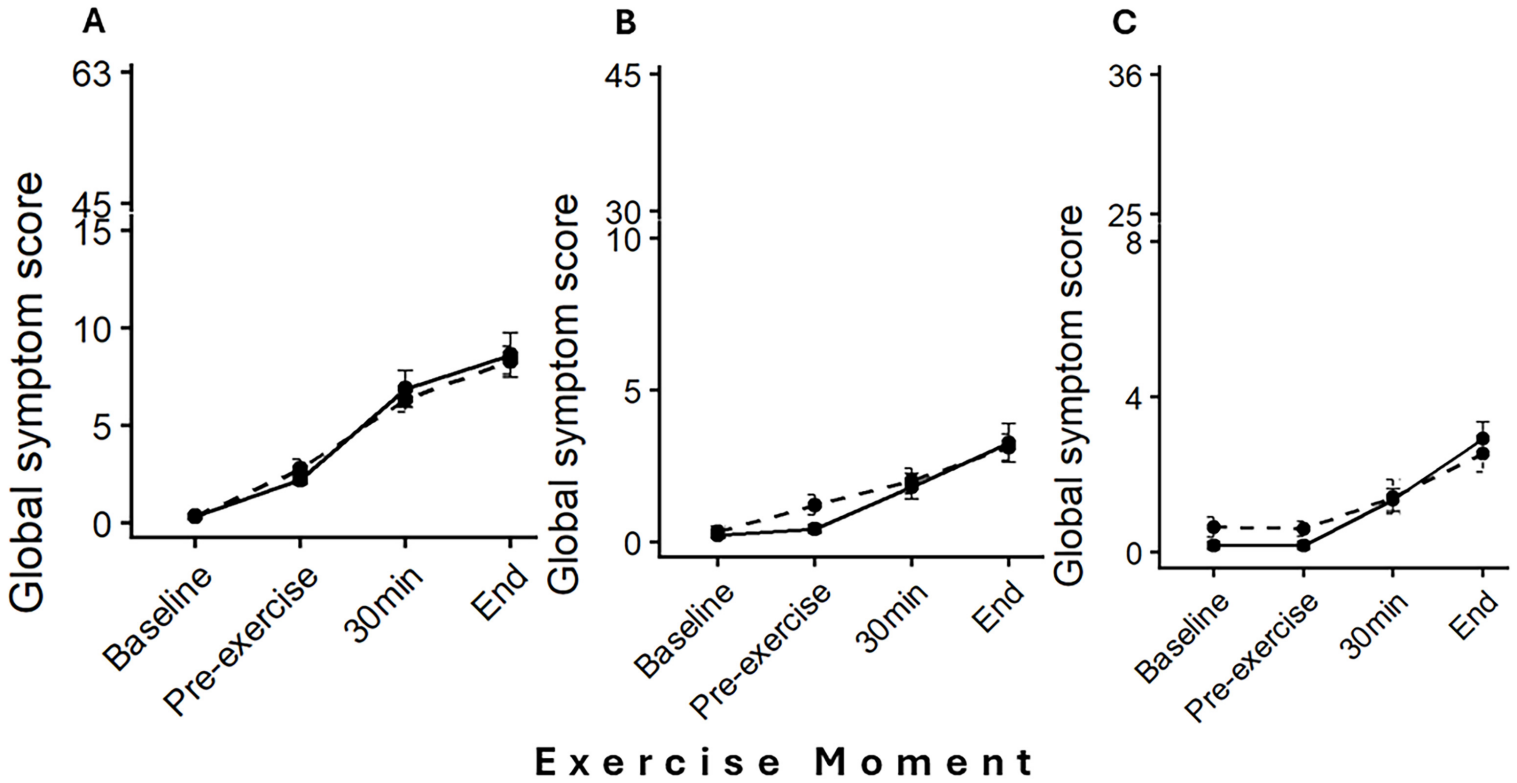
Mean global symptom score (Mean score ± SE) across exercise moments (baseline, pre-exercise, 30 min, and end of exercise) for each anatomical section: **(A)** Upper GI tract, **(B)** Lower GI tract, and **(C)** Systemic. Treadmill running (T) is represented by a solid black line with circles, and Cycling (C) by a dashed black line with circles. Each panel uses a section-specific broken Y-axis, scaled to reflect the maximum possible symptom score for that section, allowing appropriate visualization of symptom severity across sections.

Treadmill running and cycling produced similar overall GI symptom burden. The cumulative ordinal models revealed no significant effect of exercise modality on upper GI (OR = 1.01, 95% CI: 0.62–1.65; *p* = 0.972), lower GI (OR = 1.30, 95% CI: 0.76–2.22; *p* = 0.339), or systemic symptoms (OR = 1.22, 95% CI: 0.71–2.12; *p* = 0.475). Both modalities produced nearly identical temporal patterns, with symptoms increasing progressively throughout exercise (Figure 2 and Table 1). The most pronounced increases occurred in the upper GI tract, where prevalence reached ≥ 92.9% by exercise completion in both conditions, with median total scores of 7.50 [IQR: 4.00–11.00] (T) and 8.50 [IQR: 5.00–11.25] (C).

**TABLE 1:** Gastrointestinal symptom scores by region and time point.

Region (possible range)	Time	Treadmill Median (IQR)	Cycling Median (IQR)	*p*-value
Upper GI (0–63)	Baseline	0.00 [0.00–0.00]	0.00 [0.00–0.00]	1.000
	Pre-exercise	2.00 [1.00–3.00]	2.00 [0.00–4.00]	0.306
	30 min	5.00 [4.00–9.25]	6.00 [4.00–7.00]	0.504
	End of exercise	7.50 [4.00–11.00]	8.50 [5.00–11.25]	0.796
Lower GI (0–45)	Baseline	0.00 [0.00–0.00]	0.00 [0.00–0.00]	0.663
	Pre-exercise	0.00 [0.00–1.00]	0.00 [0.00–1.50]	0.058
	30 min	1.00 [0.00–2.25]	1.00 [0.00–3.00]	0.758
	End of exercise	2.50 [0.00–5.25]	4.00 [1.00–5.00]	0.833
Systemic (0–36)	Baseline	0.00 [0.00–0.00]	0.00 [0.00–1.00]	0.125
	Pre-exercise	0.00 [0.00–0.00]	0.00 [0.00–1.00]	0.069
	30 min	0.50 [0.00–3.00]	0.00 [0.00–2.25]	0.871
	End of exercise	3.00 [0.75–5.00]	2.50 [0.00–4.00]	0.459

Values are median [IQR]. IQR = interquartile range (25th–75th percentile). p-values represent comparisons between modalities at each time point; Baseline and Pre-exercise values are descriptive only. No statistically significant differences were observed between modalities at any time point (all *p* > 0.05).

Equivalence testing (TOST, ± 0.6 SD bounds) confirmed equivalence of total symptom scores between modalities for upper GI (p_TOST = 0.010) and systemic regions (p_TOST = 0.026). Lower GI scores were inconclusive (p_TOST = 0.056). Symptom counts did not reach equivalence in any region (all p_TOST ≥ 0.074).

Although aggregate symptom scores did not differ between modalities, several individual symptoms were more frequent during cycling than treadmill running. Heartburn/acidity was significantly more frequent during cycling (OR = 7.40, 95% CI: 2.16–25.39; *p* = 0.001), as was flatulence (OR = 2.45, 95% CI: 1.22–4.92; *p* = 0.012), defecation emergencies (OR = 231.40, 95% CI: 4.58–11698.59; *p* = 0.007), and headache presence (OR = 2.38, 95% CI: 1.02–5.55; *p* = 0.044). Among participants who reported headache, however, severity did not differ significantly between modalities (*p* = 0.997). These individual differences did not substantially affect overall symptom burden, likely because they represented a small subset of the total symptoms assessed and their absolute prevalences remained relatively low (Table 2). All other symptoms—including abdominal distension, lateral abdominal pain, intestinal cramps, nausea, reflux, urinary urgency, altered saliva consistency, eructation, and muscle cramps—showed no differences between modalities (all *p* ≥ 0.11).

**TABLE 2:** Prevalence of individual gastrointestinal and systemic symptoms by exercise modality.

Symptom	Treadmill (End) n/N (%)	Cycling (End) n/N (%)	*p*-value (Condition)
Symptoms differing between modalities			
Defecation urgency	1/28 (3.6%)	3/28 (10.7%)	0.007**
Flatulence	12/28 (42.9%)	17/28 (60.7%)	0.012*
Heartburn/acidity	2/28 (7.1%)	8/28 (28.6%)	0.001**
Headache	7/28 (25.0%)	10/28 (35.7%)	0.044*
Symptoms with no differences			
Abdominal distension	10/28 (35.7%)	11/28 (39.3%)	1.000
Lateral abdominal pain	7/28 (25.0%)	5/28 (17.9%)	0.111
Intestinal cramps	5/28 (17.9%)	5/28 (17.9%)	0.293
Nausea	7/28 (25.0%)	8/28 (28.6%)	0.765
Reflux	11/28 (39.3%)	15/28 (53.6%)	0.326
Urinary urgency	15/28 (53.6%)	14/28 (50.0%)	0.327
Muscle cramps	10/28 (35.7%)	10/28 (35.7%)	0.413
Thickened or cut saliva	23/28 (82.1%)	24/28 (85.7%)	0.877
Belching	26/28 (92.9%)	27/28 (96.4%)	0.558
Rare symptoms (descriptive only)			
Cramps	1/28 (3.6%)	1/28 (3.6%)	–
Dizziness	1/28 (3.6%)	1/28 (3.6%)	–
Vomiting	2/28 (7.1%)	0/28 (0.0%)	–

Data are presented as number of participants with symptoms over total (n/N), with percentage in parentheses, using values at the end of exercise. *P*-values correspond to the main effect of condition (treadmill vs. cycling) derived from longitudinal models (GLMM, GLM, ordinal or hurdle models as appropriate). Statistical significance was defined as *p < 0.05 and **p < 0.01. Symptoms with very low frequency (≤ 2 cases) were analyzed descriptively only.

Symptoms increased progressively across all time points relative to baseline across all anatomical regions (all *p* ≤ 0.005), with the strongest increases observed in the upper GI tract at 30 min (OR = 241.36, 95% CI: 85.56–680.85; *p* < 0.001) and end of exercise (OR = 535.16, 95% CI: 181.48–1578.10; *p* < 0.001), reflecting the marked rise in belching, thickened saliva, and reflux during exercise.

### 3.2 Palatability

**FIGURE 3:**
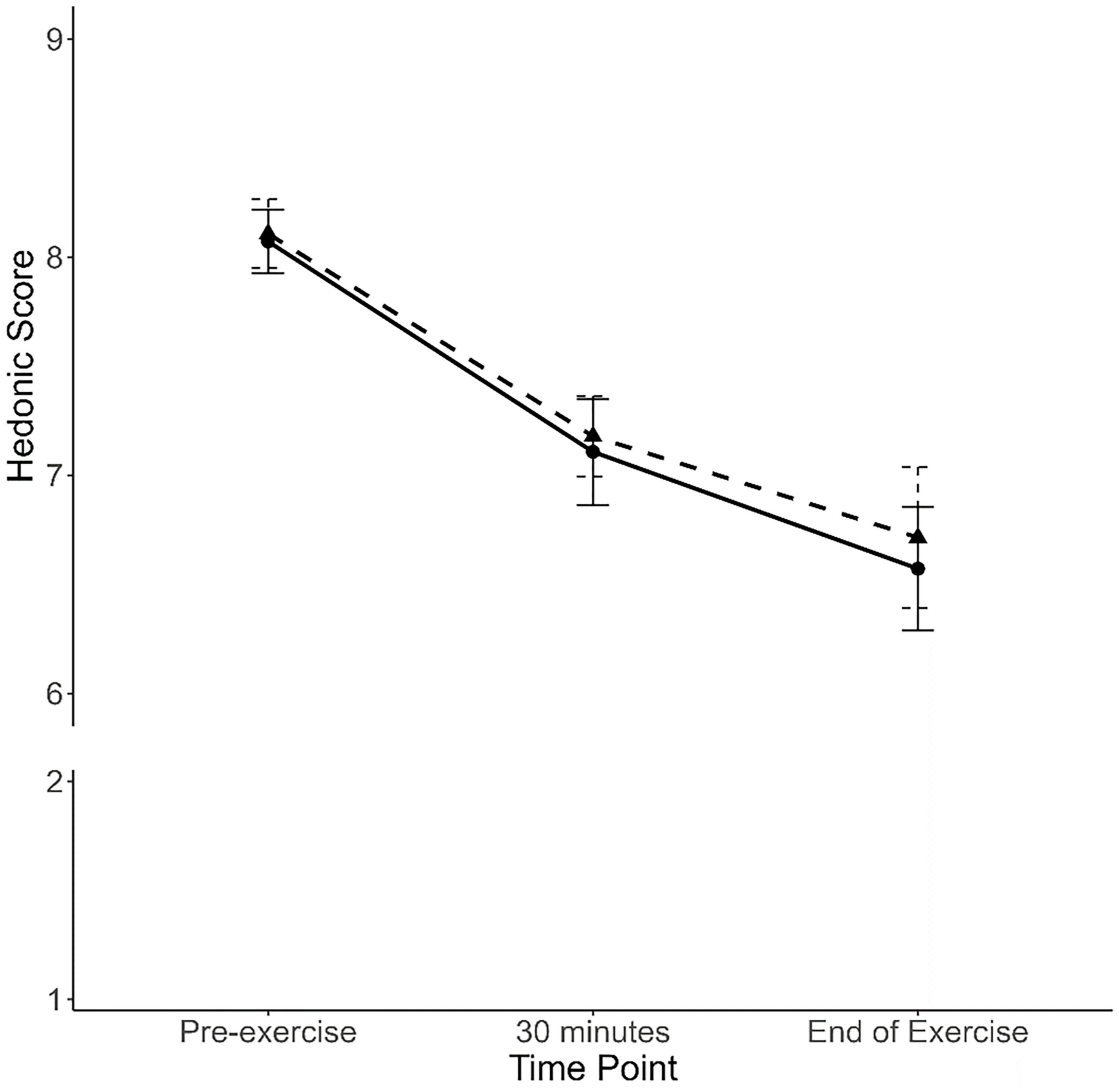
Hedonic scores (mean ± SE) for palatability at pre-exercise, 30 min, and end of exercise. Running trials are shown with a solid black line and circles, and cycling trials with a dashed black line and triangles. Note: the y-axis is broken; observed scores ranged from 6 to 9 on a 9-point scale, where 1 = extremely unpleasant and 9 = extremely pleasant.

Hedonic ratings of the lactose-free A2 milk remained high throughout both exercise protocols, declining modestly from approximately 8.1 at pre-exercise to 6.6 by the end of exercise (Figure 3). The mixed-effects beta regression confirmed a significant time effect, with lower scores at 30 min (T: 7.11 ± 1.29; C: 7.18 ± 0.98) and end of exercise (6.57 ± 1.50; 6.71 ± 1.72) compared to pre-exercise (8.07 ± 0.77; 8.11 ± 0.83; all comparisons *p* < 0.001 after Bonferroni adjustment). Exercise modality had no effect on palatability at any time point (all *p* > 0.05), and the condition × time interaction was non-significant.

### 3.3 Physiological variables

Mean heart rate was higher during treadmill running (142 ± 6 bpm) than cycling (138 ± 6 bpm; *p* = 0.006; *p*_FDR = 0.031; *d* = 0.57), as was final heart rate (146 ± 7 vs. 141 ± 5 bpm; *p* = 0.001; p_FDR = 0.014; *d* = 0.68); nevertheless, both values remained within the prescribed 70–80% HRmax target range. All other physiological and hydration variables were comparable between conditions. Sweat rate averaged 0.88 ± 0.38 L/h for T and 0.86 ± 0.38 L/h for C (*p* = 0.754), while total fluid intake was standardized at 16 mL/kg (∼1,076 mL) for both modalities. Body weight change, urine output, and initial urine specific gravity showed no significant differences between T and C (all *p* > 0.05; Table 3).

**TABLE 3:** Descriptive statistics of physiological variables by condition.

Variable	Treadmill mean ± SD	Treadmill median (IQR)	Cycling mean ± SD	Cycling median (IQR)	*p*-value
Initial body weight (kg)	67.28 ± 11.01	65.67 [61.25–73.50]	67.27 ± 11.25	65.87 [61.14–74.15]	0.962
Body weight change (kg)	0.08 ± 0.29	0.14 [0.03–0.19]	0.10 ± 0.28	0.14 [0.06–0.19]	0.604
Urine output (mL)	123.6 ± 53.4	121.8 [92.7–142.7]	115.6 ± 61.9	100.1 [69.4–154.3]	0.463
Total fluid intake (mL)	1,076 ± 176	1,051 [980–1,176]	1,076 ± 180	1,054 [978–1,186]	0.962
Initial HR (bpm)	80 ± 15	75 [69–92]	82 ± 12	83 [74–89]	0.558
Final HR (bpm)	146 ± 7	146 [140–151]	141 ± 5	141 [137–144]	0.001**
Mean HR (bpm)	142 ± 6	142 [137–147]	138 ± 6	137 [136–142]	0.006**
Initial USG	1.009 ± 0.005	1.008 [1.005–1.014]	1.012 ± 0.005	1.012 [1.008–1.016]	0.060
Sweat rate (L/h)	0.88 ± 0.38	0.81 [0.68–1.01]	0.86 ± 0.38	0.84 [0.68–0.95]	0.754

IQR, Interquartile Range; *p*-values from paired *t*-tests or Wilcoxon signed-rank tests as appropriate.

**p < 0.01 (raw); p_FDR reported for significant comparisons. d = Cohen’s d effect size.

## 4 Discussion

The primary finding of this study is that exercise modality did not meaningfully influence overall GI tolerance when exercise intensity, duration, hydration volume, and beverage composition were controlled. Contrary to our initial expectation, treadmill running and stationary cycling elicited comparable total GI symptom burden and nearly identical temporal symptom profiles. Upper GI symptom prevalence increased progressively over time in both modalities, reaching ≥ 92.9% by the end of exercise, while mean total upper GI symptom scores were similar for treadmill running (8.61 ± 5.96) and cycling (8.32 ± 3.84), indicating substantial but comparable symptom loads.

These findings contrast with the prevailing view—largely derived from field studies in endurance runners and triathletes—that running induces more frequent and severe GI complaints than cycling due to greater mechanical impact and higher rates of splanchnic hypoperfusion and exercise-induced GI injury (1–3, 25). For example, long-distance runners report a higher prevalence of lower GI symptoms than cyclists, and marathon participation has been associated with GI distress in 25–52% of competitors (1, 3).

Several methodological features of field studies likely amplify apparent modality-related differences. Exercise intensity, duration, and thermal strain are rarely standardized, with self-selected pacing, longer running durations, and hotter environmental conditions increasing dehydration, core temperature, and splanchnic hypoperfusion (1, 26). In addition, hydration and fueling practices differ systematically between modalities, with greater fluid and carbohydrate intake typically achieved during cycling and triathlon bike segments than during running (4). These factors complicate the isolation of mechanical impact from nutritional and metabolic stressors.

Consistent with controlled findings by Costa et al. (5), who reported comparable GI symptoms and intestinal integrity perturbations during matched-intensity running and cycling in the heat, and with Edwards et al. (7), who demonstrated that intestinal damage assessed via I-FABP—a biomarker of enterocyte injury rather than perceived symptoms—that intestinal damage was paradoxically greater during cycling than running at matched absolute V̇O_2_ (attributable to higher physiological strain during cycling rather than to modality-specific mechanical factors), the present results indicate that when workload, hydration, and nutritional intake are standardized, modality-related differences in GI tolerance are substantially attenuated. It is worth noting that GI symptoms and mucosal damage do not necessarily co-vary (7, 27), and the present study extends this body of evidence to the domain of perceived symptom tolerance during consumption of a milk-based beverage—a context not addressed in prior biomarker studies.

An additional point that warrants emphasis is that symptom severity remained low in the present study despite the moderate-to-high exercise intensity prescribed (70–80% HRmax). Prolonged submaximal exercise and greater thermoregulatory strain have been linked to more pronounced perturbations of GI integrity and higher GI symptom burden, particularly when core temperature approaches or exceeds ∼39°C (5, 26). In contrast, our standardized protocol at a lower thermal load elicited only mild symptoms across both modalities, suggesting that when beverage composition, hydration volume, and exercise load are carefully controlled, a milk-based hydration strategy can remain well tolerated even under relatively demanding endurance conditions. Although mean heart rate was higher during treadmill running than cycling (142 ± 6 vs. 138 ± 6 bpm; *p* = 0.006; p_FDR = 0.031; *d* = 0.57), and similarly for final heart rate (146 ± 7 vs. 141 ± 5 bpm; *p* = 0.001; p_FDR = 0.014; *d* = 0.68), both values remained within the prescribed 70–80% HRmax target range. The equivalent sweat rates and hydration variables between conditions further indicate that thermoregulatory and metabolic demands were comparable. The higher HR during running likely reflects the greater mechanical cost of weight-bearing locomotion relative to cycling at a matched workload, rather than a meaningfully different exercise stimulus.

Although overall GI symptom burden did not differ between modalities, specific symptoms such as heartburn/acidity, flatulence, defecation urgency, and headache differed significantly between conditions (*p* < 0.05). These differences are unlikely to be explained by absolute intra-abdominal pressure (IAP), as seated cycling produces substantially lower peak IAP than running or jumping (28). Instead, the sustained flexed seated posture during cycling may redistribute modest but continuous IAP toward abdominal and pelvic viscera, promoting gas pooling, altered colonic motility, and transient gastroesophageal reflux.

Importantly, these symptoms accounted for only a small subset of all assessed symptoms and showed modest absolute prevalences, resulting in no meaningful increase in composite lower GI or systemic symptom scores. Collectively, these observations suggest that posture-related IAP distribution may shape specific symptom profiles without substantially impairing overall GI tolerance under controlled nutritional and metabolic loads.

Across both exercise modalities, the most robust effect observed was time. Upper GI symptoms increased markedly from pre-exercise to the end of exercise (ORs ranging from 16.71 to 535.16; *p* < 0.001), with similar temporal patterns observed for lower GI and systemic symptoms. Additionally, symptom severity continued to rise from 30 min to exercise termination across all domains (*p* < 0.05). This progressive increase likely reflects the combined influence of sustained gastric volume (≈16 mL⋅kg^–1^ over 70 min) and exercise-induced reductions in splanchnic blood flow, rather than exercise modality *per se*.

Although gastric emptying was not directly assessed, moderate-to-high intensity exercise (≥ 60–70% V̇O_2_max) combined with a beverage containing protein and fat would be expected to delay gastric clearance (11, 29), consistent with the progressive rise in upper GI symptoms. The overlapping temporal symptom trajectories for running and cycling further support the notion that cumulative metabolic and volumetric stress, rather than mechanical impact, is the primary driver of symptom development under these conditions.

The present findings extend prior work demonstrating the feasibility of milk-based hydration strategies during exercise (9, 11, 13, 30). While previous controlled studies have largely focused on cycling, this study provides the first direct evidence that low-fat, lactose-free A2 milk is similarly tolerated during treadmill running under standardized conditions.

Despite progressive increases in GI symptoms over time, symptom severity remained low, no participants withdrew due to GI distress, and palatability ratings remained within an acceptable range (group mean ≥ 6) in both modalities. The lactose-free formulation and exclusive A2 β-casein content likely contributed to favorable tolerance by minimizing lactose malabsorption and A1 β-casein–related intolerance (10, 31). This interpretation is further supported by recent controlled evidence showing improved gastrointestinal tolerance with lactose-free A2 milk compared with conventional formulations, particularly in individuals susceptible to dairy-related GI discomfort (15).

From an applied perspective, these findings suggest that GI tolerance assessed during cycling-based laboratory protocols may reasonably translate to running when exercise intensity, hydration volume, and beverage composition are standardized. Low-fat, lactose-free milk should not be considered inherently unsuitable for running-based exercise solely due to concerns about mechanical impact. For multi-disciplinary athletes, such as triathletes, and for practitioners designing endurance nutrition strategies, beverage composition and individual tolerance appear to be more critical determinants of GI comfort than exercise modality *per se*. As with all hydration strategies, individual experimentation during training remains essential given substantial inter-individual variability in GI sensitivity. The present study assessed acute GI responses during a single 60-min exercise session and therefore cannot address long-term tolerability, gut adaptation following repeated beverage exposure, or the GI demands of prolonged events such as marathons or ultramarathons, where greater cumulative fluid volumes, sustained splanchnic hypoperfusion, and progressive thermal strain may produce substantially different responses; these questions warrant dedicated longitudinal and field-based investigation.

Several considerations should be noted when interpreting these findings. The study was conducted under controlled laboratory conditions with a fixed 60-min exercise duration, which strengthened internal validity but may not fully reflect real-world endurance settings or events exceeding 90 min, where cumulative fluid volume, greater thermal strain, and progressive fatigue may produce substantially different GI responses. Exercise intensity was prescribed using age-predicted HRmax rather than directly measured V̇O_2_peak, and although RPE was monitored verbally, neither RPE values, V̇O_2_, nor absolute workload parameters (treadmill speed and cycling power output) were formally recorded; future studies employing V̇O_2_peak-based prescription, systematic perceptual monitoring, and standardized absolute workload recording would allow more precise physiological characterization and improve comparability across modalities. Pre-trial dietary intake was standardized through written and verbal instructions but not objectively verified, which limits the ability to fully exclude fermentable carbohydrate intake as a confounding variable. The GI symptom scale has not undergone formal psychometric validation, no biomarkers of intestinal integrity (e.g., I-FABP) were measured, and no direct assessment of total body water was included; additionally, GI symptoms were not monitored beyond the immediate post-exercise period. Future work combining validated symptom instruments with biochemical markers, hydration indices, and extended post-exercise follow-up would strengthen both mechanistic interpretation and assessment of functional relevance. The equivalence bounds were based on a power-derived smallest effect size of interest (24) rather than a formally validated minimal clinically important difference, as none currently exists for exercise-induced GI symptom scales. Finally, the sample consisted of young, physically active individuals familiar with both modalities, and findings apply to a single well-characterized beverage; the small number of female participants (*n* = 6) precluded sex-stratified analyses, and generalization to other populations, dairy or non-dairy beverages, or longer events requires further investigation.

## 5 Conclusion

When exercise intensity, duration, hydration volume, and beverage composition were controlled, treadmill running and stationary cycling produced comparable GI symptom burden and temporal profiles. Contrary to common assumptions derived from field studies, exercise modality alone did not meaningfully influence overall GI tolerance, indicating that mechanical impact during running plays a limited role under standardized conditions.

Although cycling was associated with modest increases in select symptoms, these differences did not affect composite GI scores and are more plausibly explained by posture-related factors than by absolute mechanical stress. Low-fat, lactose-free A2 milk was well tolerated in both modalities, maintaining acceptable palatability and hydration without exercise-limiting GI distress. Collectively, these findings challenge the notion that dairy-based beverages are unsuitable for running and underscore the importance of controlling physiological and nutritional variables when evaluating GI tolerance during endurance exercise. These conclusions apply specifically to approximately 60 min of continuous endurance exercise at moderate-to-high intensity under controlled laboratory conditions and should not be generalized to events of greater duration, higher intensity, or non-thermoneutral field settings without further investigation.

## Data Availability

The datasets presented in this study can be found in online repositories. The names of the repository/repositories and accession number(s) can be found at: https://doi.org/10.5281/zenodo.17517219.

## References

[1] Rehrer N Janssen G Brouns F Saris W. Fluid intake and gastrointestinal problems in runners competing in a 25-km race and a Marathon*. *Int J Sports Med.* (1989) 10:S22–5. 10.1055/s-2007-1024950 2744925

[2] Peters HP van Schelven FW Verstappen PA de Boer RW Bol E Erich WB et al. Gastrointestinal problems as a function of carbohydrate supplements and mode of exercise. *Med Sci Sports Exerc.* (1993) 25:1211–24. 10.1249/00005768-199311000-00003 8289607

[3] Peters HP Bos M Seebregts L Akkermans L van Berge Henegouwen G Bol E et al. Gastrointestinal symptoms in long-distance runners, cyclists, and triathletes: prevalence, medication, and etiology. *Am Coll Gastroenterol.* (1999) 94:1570–81. 10.1111/j.1572-0241.1999.01147.x 10364027

[4] Pfeiffer B Stellingwerff T Hodgson AB Randell R Pöttgen K Res P et al. Nutritional intake and gastrointestinal problems during competitive endurance events. *Med Sci Sports Exerc.* (2012) 44:344–51. 10.1249/MSS.0b013e31822dc809 21775906

[5] Costa RJ Mika AS McCubbin AJ. The impact of exercise modality on exercise-induced gastrointestinal syndrome and associated gastrointestinal symptoms. *J Sci Med Sport.* (2022) 25:788–93. 10.1016/j.jsams.2022.07.003 35868987

[6] De Oliveira EP Burini RC. The impact of physical exercise on the gastrointestinal tract. *Curr Opin Clin Nutr Metab Care.* (2009) 12:533–8. 10.1097/MCO.0b013e32832e6776 19535976

[7] Edwards KH Ahuja KD Watson G Dowling C Musgrave H Reyes J et al. The influence of exercise intensity and exercise mode on gastrointestinal damage. *Appl Physiol Nutr Metab.* (2021) 46:1105–10. 10.1139/apnm-2020-0883 33725465

[8] de Oliveira EP Burini RC Jeukendrup A. Gastrointestinal complaints during exercise: prevalence, etiology, and nutritional recommendations. *Sports Med.* (2014) 44:79–85. 10.1007/s40279-014-0153-2 24791919 PMC4008808

[9] Pegoretti C Antunes AEC Manchado-Gobatto F de B Capitani CD. Milk: an alternative beverage for hydration? *Food Nutr Sci.* (2015) 06:547–54. 10.4236/fns.2015.66057

[10] Desbrow B Jansen S Barrett A Leveritt MD Irwin C. Comparing the rehydration potential of different milk-based drinks to a carbohydrate–electrolyte beverage. *Appl Physiol Nutr Metab.* (2014) 39:1366–72. 10.1139/apnm-2014-0174 25315686

[11] Aragón-Vargas LF Garzón-Mosquera JC Montoya-Arroyo JA. Skimmed, lactose-free milk ingestion postexercise: rehydration effectiveness and gastrointestinal disturbances versus water and a sports drink in physically active people. *Int J Sport Nutr Exerc Metab.* (2024) 1:1–9. 10.1123/ijsnem.2023-0253 38789098

[12] Maughan RJ Watson P Cordery PA Walsh NP Oliver SJ Dolci A et al. A randomized trial to assess the potential of different beverages to affect hydration status: development of a beverage hydration index. *Am J Clin Nutr.* (2016) 103:717–23. 10.3945/ajcn.115.114769 26702122

[13] Aragón-Vargas LF Garzón-Mosquera JC Montoya-Arroyo JA. Voluntary hydration with skimmed lactose-free milk during exercise in the heat: exploring effectiveness and tolerance. *Nutrients.* (2023) 15:2069. 10.3390/nu15092069 37432231 PMC10181011

[14] Boro TL Rezaei S Kavoura IE Kooima P Wasserbeck A Schott KD et al. Improved postexercise rehydration with a milk permeate-based sports drink. *Int J Sport Nutr Exerc Metab.* (2025) 1:30–41. 10.1123/ijsnem.2025-0067 41297533

[15] Robinson LA Cavanah AM Lennon S Mattingly ML Anglin DA Boersma MD et al. Lactase-treated A2 milk as a feasible conventional milk alternative: results of a randomized controlled crossover trial to assess tolerance, gastrointestinal distress, and preference for milks varying in casein types and lactose content. *Nutrients.* (2025) 17:1946. 10.3390/nu17121946 40573057 PMC12196342

[16] Warburton DE Jamnik V Bredin SS Shephard RJ Gledhill N. The 2020 physical activity readiness questionnaire for everyone (PAR-Q+) and electronic physical activity readiness medical examination (ePARmed-X+): 2020 PAR-Q+. *Health Fitness J Can.* (2019) 12:58–61. 10.14288/hfjc.v12i4.295

[17] Armstrong LE Pumerantz AC Fiala KA Roti MW Kavouras SA Casa DJ et al. (2010). Human hydration indices: acute and longitudinal reference values. *Int J Sport Nutr Exerc Metab.* 20:145–153. 10.1123/ijsnem.20.2.145 20479488

[18] Nicolas L Marquilly C O’Mahony M. The 9-point hedonic scale: Are words and numbers compatible? *Food Quality Prefer.* (2010) 21:1008–15. 10.1016/j.foodqual.2010.05.017

[19] Tanaka H Monahan KD Seals DR. Age-predicted maximal heart rate revisited. *J Am Coll Cardiol.* (2001) 37:153–6. 10.1016/s0735-1097(00)01054-8 11153730

[20] Olson RD Vaux-Bjerke A Quam JB Piercy KL Troiano RP George SM et al. *Physical Activity Guidelines for Americans.* (2018). Available online at: https://n2t.net/ark:/21207/NADAR.v3i166.48 (accessed March 15, 2023).

[21] Achten J Jeukendrup AE. Heart rate monitoring. *Sports Med.* (2003) 33:517–38. 10.2165/00007256-200333070-00004 12762827

[22] Nakagata T Murade S Katamoto S Naito H. Heart rate responses and exercise intensity during a prolonged 4-hour individual cycling race among japanese recreational cyclists. *Sports.* (2019) 7:109. 10.3390/sports7050109 31075968 PMC6572307

[23] Borg GA. Psychophysical bases of perceived exertion. *Med Sci Sports Exerc.* (1982) 14:377–81. 10.1249/00005768-198205000-00012 7154893

[24] Lakens D. Equivalence tests: a practical primer for t tests, correlations, and meta-analyses. *Soc Psychol Pers Sci.* (2017) 8:355–62. 10.1177/1948550617697177 28736600 PMC5502906

[25] Al-Beltagi M Saeed NK Bediwy AS El-Sawaf Y Elbatarny A Elbeltagi R. Exploring the gut-exercise link: a systematic review of gastrointestinal disorders in physical activity. *World J Gastroenterol.* (2025) 31:106835. 10.3748/wjg.v31.i22.106835 40539198 PMC12175863

[26] Snipe RMJ Khoo A Kitic CM Gibson PR Costa RJS. The impact of exertional-heat stress on gastrointestinal integrity, gastrointestinal symptoms, systemic endotoxin and cytokine profile. *Eur J Appl Physiol.* (2018) 118:389–400. 10.1007/s00421-017-3781-z 29234915

[27] Karhu E Forsgård RA Alanko L Alfthan H Pussinen P Hämäläinen E et al. Exercise and gastrointestinal symptoms: running-induced changes in intestinal permeability and markers of gastrointestinal function in asymptomatic and symptomatic runners. *Eur J Appl Physiol.* (2017) 117:2519–26. 10.1007/s00421-017-3739-1 29032392 PMC5694518

[28] Dietze-Hermosa M Hitchcock R Nygaard IE Shaw JM. Intra-abdominal pressure and pelvic floor health: should we be thinking about this relationship differently? *Urogynecology.* (2020) 26:409. 10.1097/SPV.0000000000000799 32574030 PMC8974352

[29] Jeukendrup A Moseley L. Multiple transportable carbohydrates enhance gastric emptying and fluid delivery. *Scand J Med Sci Sports.* (2010) 20:112–21. 10.1111/j.1600-0838.2008.00862.x 19000102

[30] Shirreffs SM Watson P Maughan RJ. Milk as an effective post-exercise rehydration drink. *Br J Nutr.* (2007) 98:173–80. 10.1017/S0007114507695543 17459189

[31] Brooke-Taylor S Dwyer K Woodford K Kost N. Systematic review of the gastrointestinal effects of A1 compared with A2 β-Casein. *Adv Nutr.* (2017) 8:739–48. 10.3945/an.116.013953 28916574 PMC5593102

