# Correction: Gastrointestinal tolerance to a standardized milk-based hydration strategy is similar across exercise modalities

**DOI:** 10.3389/fnut.2026.1866673

**Published:** 2026-05-11

**Authors:** Frontiers Production Office

**Affiliations:** Frontiers Media SA, Lausanne, Switzerland

**Keywords:** endurance exercise, exercise modality, gastrointestinal symptoms, hydration strategy, lactose-free A2 milk

There were numerous corrections not included in the published article.

There was a mistake in Figure 2 as published. An updated Figure was not published.

The corrected Figure 2 appears below.

**Figure 2:**
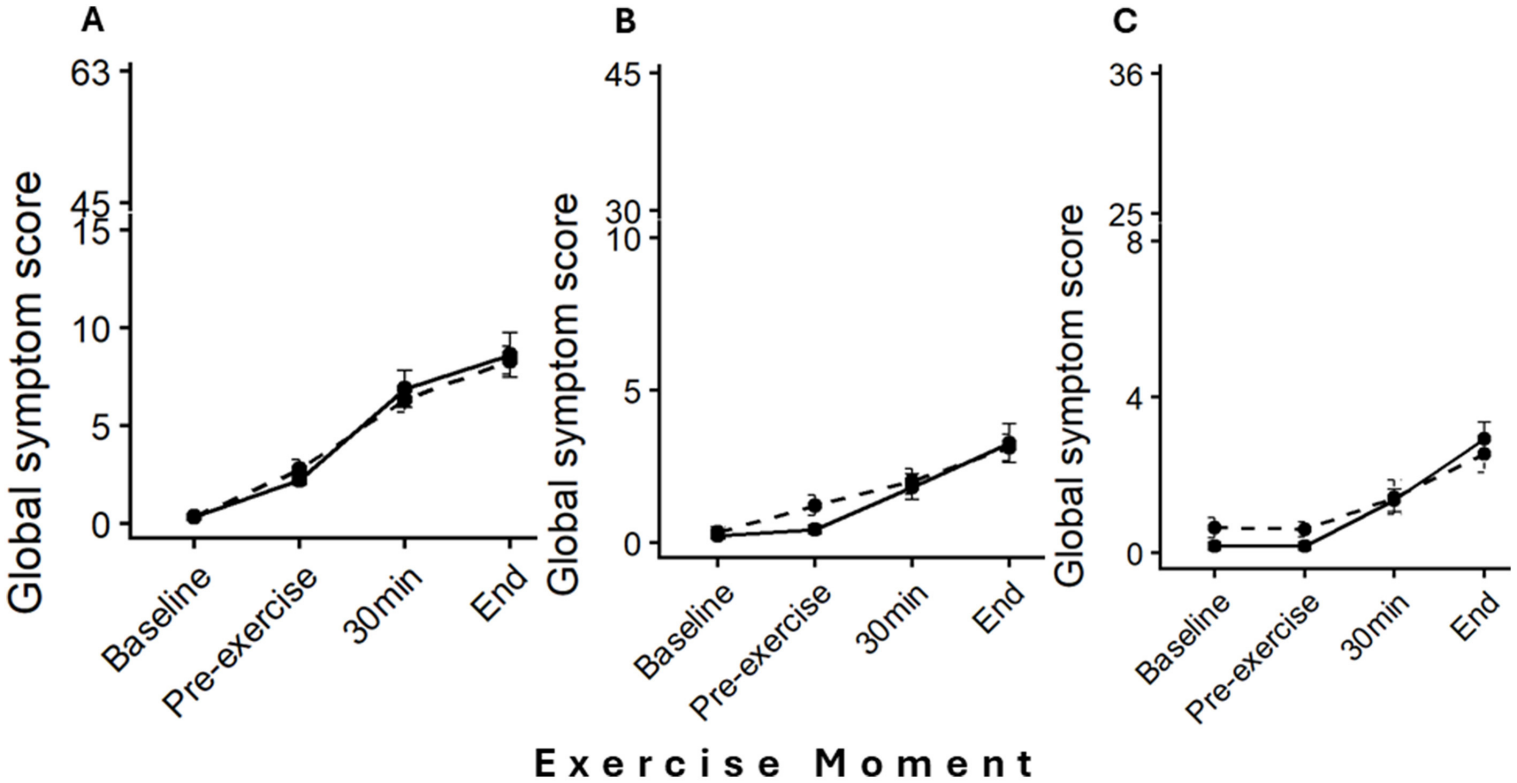
Mean global symptom score (Mean score ± SE) across exercise moments (baseline, pre-exercise, 30min, and end of exercise) for each anatomical section: **(A)** Upper GI tract, **(B)** Lower GI tract, and **(C)** Systemic. Treadmill running (T) is represented by a solid black line with circles, and Cycling (C) by a dashed black line with circles. Each panel uses a section-specific broken Y-axis, scaled to reflect the maximum possible symptom score for that section, allowing appropriate visualization of symptom severity across sections.

The original version of this article has been updated.

## Generative AI statement

Any alternative text (alt text) provided alongside figures in this article has been generated by Frontiers with the support of artificial intelligence and reasonable efforts have been made to ensure accuracy, including review by the authors wherever possible. If you identify any issues, please contact us.

